# The Humoral Immune Response against Human Endogenous Retroviruses in Celiac Disease: A Case–Control Study

**DOI:** 10.3390/biomedicines12081811

**Published:** 2024-08-09

**Authors:** Marco Bo, Roberto Manetti, Maria Luigia Biggio, Leonardo A. Sechi

**Affiliations:** 1 Department of Biomedical Sciences, Section of Microbiology and Virology, University of Sassari, Viale San Pietro 43b, 07100 Sassari, Italy; m.bo4@studenti.uniss.it; 2 Struttura Complessa Microbiologia e Virologia, Azienda Ospedaliera Universitaria Sassari, 07100 Sassari, Italy; 3 Department of Medicine, Surgery and Pharmacology, University of Sassari, 07100 Sassari, Italy; rmanetti@uniss.it (R.M.);

**Keywords:** celiac disease, HERV-K, HERV-H, HERV-W, antigenic peptides, humoral immune response

## Abstract

Background: Celiac disease (CD) is an immune-mediated disease characterized by disruptions of the small intestine. Factors such as viral and bacterial infections can trigger CD. Recently, the reactivation of Human Endogenous Retroviruses (HERVs) has also been implicated, but little is known about their specific role in patients with celiac disease. Methods: The purpose of this study is to explore the humoral immune response mounted against epitopes derived from the envelope portion of three families of HERVs (HERV-K, HERV-H, and HERV-W) in CD patients. Reactivity against the HERV-K, HERV-H, and HERV-W env-su peptides was tested by indirect ELISAs in plasma of 40 patients with celiac disease and 41 age-matched healthy subjects (HCs). Results: HERV-K, HERV-H, and HERV-W env-su peptides triggered different antibody responses in CD patients compared to HCs, with a stronger reactivity (*p* = 0.0001). Conclusions: Present results show, for the first time, that epitopes of HERV-K, HERV-H, and HERV-W are more recognized in patients with CD. Taking into consideration their proinflammatory and autoimmune features, this might suggest that HERVs may contribute to the development of CD or its exacerbation in genetically predisposed subjects. Finally, to elucidate the interplay between gut inflammation and HERVs during the inflammatory process, further studies are required. Those investigations should focus on the expression levels of HERVs and their relationship with the immune response, specifically examining anti-transglutaminase 2 (TG2) antibody levels under both gluten-free and gluten-containing dietary conditions.

## 1. Introduction

Celiac disease (CD) is a chronic, multi-organ cell-immune-mediated disease triggered by the ingestion of gluten. In CD, the immune system aberrantly attacks the healthy tissue, leading to damage to the surface of the small bowel and a reduction in nutrient absorbance [1,2,3,4]. The only effective treatment for CD is a strict, lifelong gluten-free diet (GFD). The precise etiology of the immune response remains unclear, but is thought to involve a combination of genetic predisposition and environmental factors precipitated by the ingestion of gluten [5]. CD is strongly associated with HLA-DQ2 (α1*0501, β1*0201) and DQ8 [6,7]. Crucially, HLA genetic testing has a significant ability to exclude the presence of CD; however, its ability to confirm the presence of CD is limited [8]. Gluten, a water-insoluble protein found in wheat, rye, and barley, is the primary environmental trigger for this condition. The clinical profile of CD is highly variable, characterized by a combination of intestinal and/or extra-intestinal symptoms. Following gluten ingestion, patients may experience a range of gastrointestinal symptoms, such as stomach aches, abdominal distension, diarrhea, constipation, tiredness, loss of appetite, weight loss, and neuropathy [1]. Factors such as infection, drugs, alpha interferons, and surgery have also been shown to play a role in triggering the disease [9]. Interestingly, Human Endogenous Retroviruses (HERVs) may also play a role in the disease. Over the past three decades, many researchers have highlighted the role of HERVs as potential contributors to different cell-mediated diseases and autoimmune diseases, particularly as incidences of autoimmunity and cancer are increasing worldwide. A range of factors, including microbiome, viruses, and others, could act through the innate component of the immune system, including TLRs [10,11] and innate lymphoid cells [12], potentially mediating the effects of HERVs in patients with CD. The development of CD is characterized by the involvement of different cell types and by the presence of serum autoantibodies (auto-Abs), which are crucial for diagnosis. Anti-transglutaminase 2 (TG2) antibodies (Abs) are directed against the major CD autoantigen, and it has been found that a high concentration of serum IgA anti-TG2 is strongly associated with the presence of villous atrophy in small-intestinal biopsies [13]. The inflammatory response in patients with CD is driven by CD4 T helper cell type 1 (Th1), consequently to dietary gluten. In addition, interferon alpha (IFN-α), a cytokine capable of promoting IFN-gamma (IFN-γ) synthesis, is highlighted as the principal factor implicated in the development of Th1-mediated immune diseases [14,15,16]. Type I interferons (IFN-Is), produced by various cell types, contribute to antiviral defense and modulate both the innate and adaptive immune responses [17,18]. In this context, it has been observed that HERVs can reactivate after a viral infection with different mechanisms and have a protective or negative effect in the inflammatory process, as shown in neurological, rheumatological, and metabolic diseases, and cancer. Conversely, in a small percentage of cases, the increase in autoantibodies anti-HERVs, and anti-IFN-I may be responsible for triggering damage to the body instead of protecting it against infectious disease.

Little information on the possible role of HERVs and anti-IFN-I Abs in patients with CD is available in the literature.

A comprehensive search strategy was devised and employed for the literature databases PubMed, Scopus, and Web of Science. The keywords “celiac disease and HERVs” were used to refine the scope of the literature identified in our initial searches, and we found only one research article in this field [19]. To explore the possible role of HERVs and IFN-Is in patients with CD, we investigated the possibility that neutralizing anti-IFN-I Abs may be responsible for bind blocking type I interferon, therefore delaying or forbidding the activation of the antiviral response. Finally, the humoral immune responses to three families of HERVs have been studied to assess the serological levels of HERV-K, HERV-H, and HERV-W peptides, including a correlational analysis between anti-IFN-Is and anti-TG2 Ab levels in plasma of children, adolescents, adults with CD and healthy subjects (HCs).

## 2. Materials and Methods

### 2.1. Study Design, Population, and Enrollment Criteria of the Study

This study investigates the specific antibody responses to HERV antigens and IFN-α to explore the role of HERVs in patients with CD in comparison to HCs. Peripheral whole blood was collected from 81 patients in accordance with the Declaration of Helsinki and the study protocols were approved by the Institutional Review Board (IRB) of Azienda Ospedaliero-Universitaria of Sassari, Italy (PG219/CE/2 2005). Only celiac patients satisfying disease-specific classification criteria, according to the European guidelines for the diagnosis and management of CD [20], were enrolled in the study. Collected data relative to CD patients included antibody-positive to anti-transglutaminase 2 (TG2) [21], (HLA)-DQ2 and HLA-DQ8 [6], and anti-endomysium Abs.

Forty consecutive unselected Sardinian patients with CD referred to the out-patient clinic of the Allergology Unit at the Department of Medicine, Surgery and Pharmacology at the University of Sassari in Sassari, Italy, were recruited from January 2012 to December 2012 in this case–control study.

Forty-one healthy sex- and age-matched subjects who were tested at the Blood Transfusion Centre of Sassari and from the Unit of Child Neuropsychiatry from the Department of Medical, Surgical and Pharmacology at the University of Sassari in Sassari, Italy, were selected for a routine laboratory examination and were enrolled in the study as healthy subjects (HCs).

Informed consent was obtained from all subjects involved in the study. Following are the exclusion criteria: malformations or syndromes, chronic autoimmune and immune-mediated diseases (e.g., Type 1 Diabetes (T1D), Multiple Sclerosis (MS), Systemics Lupus Erythematosus (SLE), and Rheumatoid Arthritis (RA), particularly if under specific treatment), endocrine diseases (including diabetes and thyroidopathy), ongoing infections, and congenital deafness.

All patients and controls lived in Sardinia. To preserve the quality and safety of the blood, all plasma samples were immediately transferred into clean polypropylene tubes using a Pasteur pipette, apportioned into 0.5 mL aliquots, and stored in a −80 °C freezer.

### 2.2. Blood Cell Separation and Antigens

Plasma was isolated from heparinized peripheral blood samples by layering on Ficoll (Sigma-Aldrich, St. Louis, MO, USA), and synthetic peptides derived from HERV-K, HERV-H, and HERV-W envelope proteins were used in this study for antigenic stimulation. Peptides were synthesized commercially at 90% purity (LifeTein, South Plainfield, NJ, USA).

### 2.3. ELISA Assays

Indirect ELISA assays to detect specific antibodies (Abs) against the selected antigens included in the study (assayed at 10 µg/mL) were performed as described below. Ninety-six-well Nunc immunoplates were coated and kept overnight at a temperature of 4 °C, with 10 µg/mL of peptides diluted in a 0.05 M carbonate–bicarbonate buffer with a pH of 9.5 (Sigma, St. Louis, MO, USA). Plates were then blocked for 1 h at room temperature with 1% non-fat dried milk in Tris-buffered saline 1X (TBS 1X-bloching solution) (Sigma) and washed twice with TBS containing 0.05% Tween-20 (TBS-T). An amount of 5 µL of the plasma samples was subsequently added to 95 µL of TBS-1X containing 1% non-fat dried milk for 2 h at room temperature. After five washes in TBS-T, 100 µL of alkaline phosphatase-conjugated goat anti-human immunoglobulin G polyclonal antibodies (1:1000; Sigma) was added for a duration of 1 h at room temperature. Plates were again washed five times in TBS-T, and para nitrophenylphosphate (Sigma) was added as a substrate for alkaline phosphatase. Plates were incubated at 37 °C in the dark for 5 min and the optical density (OD) was recorded to be at a wavelength of 405 nm using a SpectraMax Plus 384 microplate reader (Molecular Devices, Sunnyvale, CA, USA). For data normalization purposes, a highly responsive serum with a maximum Ab reactivity fixed at 1.0 arbitrary unit (AU)/mL was included in all experiments involving human plasma, and the reaction was stopped after 5 min. Negative control wells were obtained by incubation of immobilized peptides with secondary Abs alone, and their mean values were subtracted from all samples. Positive control was also included in all experiments.

### 2.4. Statistical Analysis

The data were analyzed using GraphPad Prism version 8.0 software (GraphPad Software Inc., La Jolla, CA, USA). The Shapiro–Wilk normality test was used to analyze the sample distribution. The distribution of the data was not normally distributed, according to the Gaussian bell of the Shapiro–Wilk test, and did not show a significant departure from the normality for age (*p* = ns). The cut off for positivity in each assay was calculated by ROC analysis and set at >90% specificity. The sensitivity was chosen accordingly. Significant differences between the OD values of CD and HC groups were determined by Mann–Whitney U test and Fisher’s exact test. Differences with *p* < 0.05 were considered statistically significant. The Spearman correlation test was carried out on the levels of the Abs found for the peptides analyzed. The results are expressed as a mean of duplicate 405 nm OD values of three separate experiments. 

## 3. Results

### 3.1. Antibody Responses to Immunogenic Epitopes of Three Families of HERVs in Patients with Celiac Disease and in HCs

**Figure 1:**
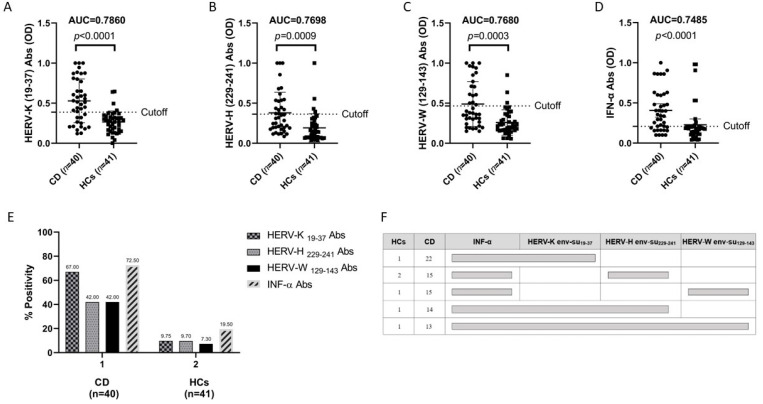
An ELISA-based analysis of Ab reactivity against viral derived peptides in the patients with celiac disease (CD) and in the healthy controls (HCs). (**A**) The sera were tested against the plate-coated HERV-K env-su_19-37_, (**B**) the HERV-H env-su_229-241_, (**C**) the HERV-W env-su_129-14_, and (**D**) INF-α peptides. The bars represent the median ± interquartile range. (**A**–**D**) The thresholds for Abs are indicated with dashed lines. The *p*-values and the AUC are indicated above the distributions. (**E**) The prevalence of the Abs tested against the HERV epitopes and INF-α in the patients with CD and in the HCs. Total percentage of Abs positivity to at least one peptide is represented by the first bar in each group. The other bars correspond to a single-peptide positivity relative to each epitope. (**F**) The prevalence of multiple Abs in patients with CD and in HCs. Seroreactivity against the INF-α antigen is compared to the humoral responses to the HERV-K, HERV-H, and HERV-W peptides. The horizontal bars indicate Abs against at least two antigens identified in the samples.

Our study’s population included 40 patients with CD (15 male, 25 female) and 41 age-matched control subjects (14 male, 26 female). The mean ages were similar in both groups: 33 ± 21.59 years in the patients with CD vs. 31.4 ± 21.16 years in the control group. Demographic, clinical, and laboratory features of the patients with celiac disease and the HCs are reported in Table 1. Abs against the HERV-K, HERV-H, and HERV-W env-su peptides were found in the plasma of the CD and HC samples, and overall, the CD subjects displayed an increased positivity to all three assessed peptides, compared to the HCs, and a high degree of statistical significance (Figure 1).

HERV-K env-su_19-37_ elicited the highest seroreactivity, accounting for 67% (n = 27) in the patients with CD and 10% (n = 4) in the HCs (AUC = 0.7860, CD versus HCs *p* < 0.0001; Figure 1A).

In regards to the HERV-H env-su_229-241_ Abs, they had a higher positivity in the sera of 42% (n = 17) of patients with CD and in only 10% (n = 4) of the HCs (AUC = 0.7698, CD versus HCs *p* =  0.0009; Figure 1B).

We found the same trend for HERV-W env-su_129-143_ peptide with a high seroreactivity among patients with CD accounting for 42% (n = 17) and 7% (n = 3) in HCs (AUC = 0.7680, CD versus HCs *p* = 0.0003; Figure 1C).

Also, we performed a stratification of the data by age group, as reported in Table 2. The analysis shows that children aged 0–11 years have a higher antibody titer to HERVs and anti-IFN-α. Conversely, there is a marked decline during adolescence, followed by a resurgence after the age of 20, which then increasingly declines after age 40.

### 3.2. Anti-HERV Profiles Relate to IFN-Alpha and Possible Synergistic Role to HERV-K, HERV-H, and HERV-W in CD in Comparison to HCs 

We investigated the immune responses against IFN-α in relation to the immune responses mounted against HERV antigens. Interferons are a family of cytokines with diverse functions during a successful host defense. Basically, the most important function of a type I IFN is to induce antiviral immunity, while IFN-γ, the only type II IFN (not investigated in this study), promotes a response to intracellular bacteria. It is well-established that IFN-α is a versatile cytokine that plays a pivotal role in the immune response, with significant therapeutic applications in infectious diseases, cancers, and autoimmune disorders. It is essential to the body’s defense against viral and bacterial infections, inhibits viral replication within host cells, enhances the degradation of viral RNA, and activates the immune cells to destroy infected cells. While type I IFNs are part of a complex cross-regulatory network, in a small percentage of cases, the increase in auto-Abs against these proteins can be harmful to the host, rather than providing them protection against infectious disease [22]. For instance, a high level of IFN auto-Abs have been observed in patients with coronavirus disease 2019 (COVID-19) admitted to the intensive care unit (ICU COVID-19 patients) in comparison to the HCs, which correlates with auto-Abs against HERV-W-env [23]. This finding is novel in that it associates IFN auto-Abs with auto-Abs against HERV-W-env, a protein recently found to be overexpressed in the lymphocytes of COVID-19 subjects and associated with severe disease and pneumonia.

Our results show an increased presence of autoantibodies (auto-Abs) against IFN-α peptide in the patients with celiac disease compared to the HCs with a *p* < 0.0001 (Figure 1D). Our hypothesis is that in some genetically predisposed individuals, for reasons that are not entirely clear, anti-IFN-α Abs develop, leading to a weak antiviral response and HERV activation. Thus, it can lead to an inefficient antiviral response that could favor viral spreading with the consequent activation of HERVs [24,25]. The presence of anti-HERV Abs is in line with what has already been published by Tovo P.A. et al., in which a higher expression of HERVs was observed in patients with celiac disease compared to the controls [26]. Our finding of anti-INF Abs correlates with the antibody responses to the HERVs, which were greater in the patients with celiac disease than in the HCs. In addition to this, we analyzed the single, double, and triple-peptide positivity among the patients with CD in comparison to the HCs and we found that 13 patients with CD were responsive to all peptides in comparison to only one individual in the HC group. This result may be indicative of a possible synergistic role of HERV-K, HERV-H, and HERV-W in patients with CD.

### 3.3. Correlation Analyses of HERV Families and Anti-TG2 Abs

**Figure 2:**
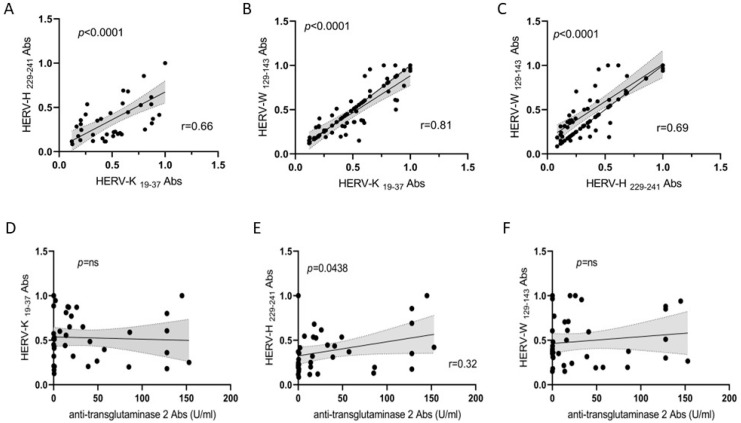
A scatter plot showing the correlations between Ab titers identifying (**A**) HERV-H env-su_229-241_ and HERV-K env-su_19-37_, (**B**) HERV-W env-su_129-143_ and HERV-K env-su_19-37_, (**C**) HERV-W env-su_129-143_ and HERV-H env-su_229-241_, (**D**) HERV-K env-su_19-37_ and α-transglutaminase, (**E**) HERV-H env-su_229-241_ and α-transglutaminase, and (**F**) HERV-W env-su_129-143_ and α-transglutaminase in 40 patients with CD. Person’s correlation was calculated through GraphPad Prism version. 8.0 software.

To define the possible associations among the antigenicity of the assessed peptides, we performed correlation analyses of the Abs positivity values in the patients with CD. The highest coefficient was obtained for HERV-K env-su peptide, followed by the HERV-H and HERV-W env-su peptides in pairwise plots, pointing to a possible synergic role of HERVs in patients with CD (Figure 2A–C, *p* < 0.0001). Also, we performed correlation analyses of the positivity values of Abs between the HERVs and the anti-TG2 Abs in the CD and HC groups (Figure 2D–F). No correlation was found between HERV-K env-su_19–37_, HERV-W env-su_129-143_, and anti-TG2 Abs (Figure 2D,F; *p* = ns). Although it was low, we found a correlation between HERV-H env-su_229-241_ and anti-TG2 Abs (Figure 2E; r = 0.32; *p* = 0.0438). However, the significance of this low correction needs to be further investigated.

### 3.4. Correlation Analysis between HERVs and IFN-α Abs in CD and HCs and between Anti-TG2 Abs and IFN-α in Patients with CD

**Figure 3:**
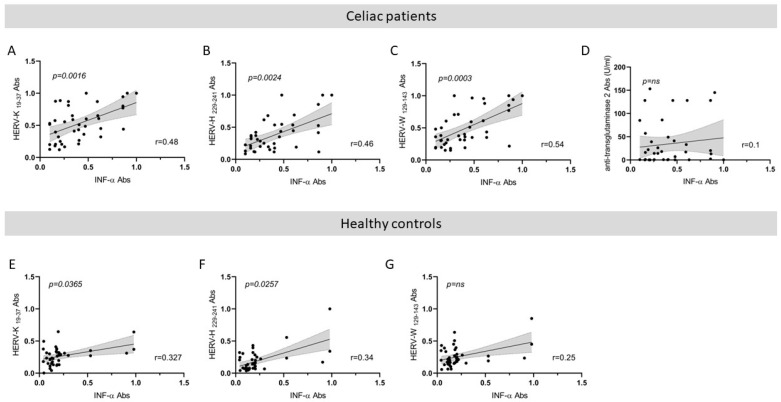
Scatter plot showing correlations between Abs titers recognizing: (**A**) HERV-K env-su19-37 and INF-α, (**B**) HERV-H env-su229-241 and INF-α, (**C**) HERV-W env-su129-143 and INF-α, (**D**) anti-transglutaminase 2 Abs and INF-α in CD patients. Scatter plot showing correlations between Abs titers recognizing: (**E**) HERV-K env-su19-37 and INF-α, (**F**) HERV-H env-su229-241 and INF-α, (**G**) HERV-W env-su129-143 and INF-α in 41 HCs. Person’s correlation was calculated through GraphPad Prism v. 8.0 software.

Finally, we performed a correlation analysis of Abs positivity values between HERVs and IFN-α both in CD and HCs observing a higher correlation in celiac patient with respect to HCs (Figure 3A–C and Figure 3E–G respectively). No correlation was found between anti-TG2 Abs and IFN-α (Figure 3D; *p* = ns).

## 4. Discussion

The increasing incidence of CD suggests that common infections before the onset of immune cell-mediated diseases could switch the immune response. Viral and bacterial infections have long been suspected to trigger an immune dysregulated response similar to an autoimmune process in patients with CD [27,28,29,30,31,32,33,34,35,36]. Over the last three decades, researchers have highlighted the role of HERVs as potential contributors toward different cell-mediated and autoimmune diseases, especially given the rising incidence of autoimmunity and cancer worldwide. HERVs, which represent 8% of our genome, are involved in different molecular mechanisms such as genes regulation, produce retroviral RNAs, and encode viral proteins that can alter both innate and adaptive immune responses. However, the role of HERVs in CD remains largely unknown.

Additionally, there is evidence that infections elicit an inflammatory response via TLR/NF-kB pathway and IRF1, leading to HERV transactivation [37]. Although HERVs protect the fetus in placental mammals, their activity has been linked to diseases like multiple sclerosis [34,35,36,37,38,39,40,41,42,43,44,45], amyotrophic lateral sclerosis [38,39,40,41,42,43,44,45,46], diabetes [47,48,49,50,51], systemic lupus erythematosus [52,53,54,55,56,57,58], rheumatoid arthritis [57,58,59,60,61,62,63], autism spectrum disorder [19,64,65,66,67,68], and cancer [69,70]. Our results show, for the first time, that the antibody responses mounted against three epitopes of HERV-K, HERV-H, and HERV-W families are significantly more elevated in the plasma of patients with celiac disease compared to the HCs. Our observation is in line with the results found by Tovo P.A. et al., in which they found that the transcriptional activity of pol genes from HERV-H, HERV-K, and HERV-W families were significantly higher in WBCs from children and adolescents with celiac disease compared to age-matched control subjects [26]. Interestingly, Table 2 shows that children with celiac disease aged 0–11 years have higher antibody titers to HERVs and anti-IFN-α compared to the healthy controls, with a decline in adolescence and a resurgence after age 20, then a decline again after age 40. This increase in the anti-HERV Abs in young children with celiac disease may be linked to a higher gluten intake, particularly in Italy, where the daily intake of wheat is 401.2 g/capita/day compared to 263.5 g in Northern Europe and 220.36 g in the USA (FAOSTAT. Available from: http://www.fao.org/faostat/en/#compare access 30 June 2024). This suggests that gluten-induced intestinal inflammation may activate HERVs. Although it is a hypothesis only, we cannot exclude the possibility that HERV activity fluctuates with the inflammatory state of the gut due to gluten ingestion, as shown by Pedretti M. et al., who observed that gastrointestinal symptoms in younger children decrease with age [71]. Even though our data do not come from a local analysis performed on stool samples or biopsies, but from a peripheral analysis of plasma, the finding that a higher presence of anti-INF-Is and anti-HERV Abs in 0–11-year-olds is consistent with the higher incidence and prevalence of intestinal disorders, such as those also observed by Pedretti M. et al.

Interestingly, the number of CD patients with plasma antibodies decreased across selected HERV epitopes K, H, and W. This suggests that various factors, including DNA methylation, deletions, and mutations, might influence HERV transcription levels and correlate with antibody production at different disease stages. Higher immunogenicity of specific peptides could also explain the variation in antibody responses, as seen in other diseases. For instance, RNA sequences of the HERV-K family have been detected in the motor neurons of ALS patients, and an increase in protein expression has been associated with neurotoxicity. Another study has highlighted that HERV-K env-su_19–37_ antibody levels significantly correlate with the clinical measures of disease severity [72]. In T1D, the HERV-W envelope protein has been identified in pancreatic cells, promoting macrophage recruitment and impairing beta-cell dysfunction, as evidenced by its inhibition of insulin secretion in primary cultures of human islets of Langerhans [48]. There is a positive correlation between the anti-HERV-W and proinsulin auto-Abs in children with T1D. Additionally, HERV-K env-su_19–37_ Abs were significantly higher in T1D patients compared to healthy controls. In RA, increases in HERV-K gene expression and viral protein transcription have been observed in patients’ peripheral blood, synovial fluid, and synoviocytes, with shared amino acid sequences between the HERV-K and host antigens, suggesting molecular mimicry may contribute to disease pathogenesis [59,63,73,74].

Interesting are the results that we found about the antibody responses mounted against IFN-α in the patients with CD. Our hypothesis is that in some genetically predisposed individuals, for reasons that are not entirely clear, anti-IFN-α Abs develop, which could lead to a weak antiviral response and HERV activation. Also, there is evidence in which a transactivation of HERVs is reported to be triggered by environmental factors such as an infection. Thus, for example, the circulation of anti-IFN-α Abs have been observed in the blood of patients with COVID-19 disease [23]. However, although it is beneficial for fighting infections and tumors, IFN-α is implicated in the development of autoimmune diseases by enhancing the presentation of self-antigens and promoting autoantibody production. Bastard et al. found that patients that lack specific IFNs may be more susceptible to infectious diseases. They discovered that almost 10% of subjects with severe COVID-19-pneumonia had high levels of neutralizing autoantibodies against type I IFN-α2 and IFN-ω [22]. These autoantibodies were absent in patients who were asymptomatic or who had a milder form of the infection, as well as in healthy individuals. It is likely that the presence of anti-IFN-I auto-Abs can interfere with the ability of IFN-Is to bind to the type I interferon receptor (IFNAR), thereby blocking the activation of the antiviral response.

In patients with celiac disease, we found a positive correlation between the HERVs and IFN-α (Figure 3), but no correlation between antibody positivity to HERVs and anti-TG2 (Figure 2). However, after stratifying by age group, we found a soft trend toward a correlation between antibody positivity to HERVs and anti-TG2 in CD patients up to 11 years of age only. Although this correlation is not statistically significant, we cannot exclude the possibility that HERVs play an important role in children compared to adults. Inflammation following gluten ingestion could lead to changes in the gut microbiota with the transactivation of HERVs. It is likely that this condition is more common in children and adolescents because they do not follow a proper diet, creating a vicious cycle that can be broken by eliminating gluten. Recent evidence has identified a new axis where bacteria influence endogenous retrovirus (ERV) expression and vice versa [75]. This connection between the microbiota and HERVs suggests a novel mechanism for immune regulation and host health, with these systems collaborating to create a beneficial immune environment. A disruption in this balance may lead to pathogenic inflammation [75].

Our results provide additional insights into CD pathogenesis, suggesting that the intestinal damage from CD cannot be ascribed solely to the gluten-driven specific immune response, but also requires an additional inflammatory response that could be deregulated by HERV transactivation. Furthermore, the significant variability in disease penetrance, severity, and presentation points to the involvement of additional genetic and environmental factors that are challenging to untangle and hierarchize. This suggests that the inflammation triggered by gluten ingestion could be intensified by HERV transactivation, creating a vicious cycle with the immune response to gluten antigens. This cycle could ultimately lead to villus damage and flattening in patients with celiac disease due to the continuous recruitment of inflammatory cells. CD4 T-cell responses to an exogenous antigen can cause an autoreactive B-cell response and facilitate the activation of intraepithelial lymphocytes to destroy intestinal epithelial cells [76,77,78].

Despite our observation of a higher immune response to HERV antigens in patients with CD, we currently do not have enough clinical and laboratory data to determine whether HERVs have the potential to be either harmful or beneficial to patients with CD. We are planning to perform further experiments with a larger group of celiac patients to gain a clearer understanding of the interaction between gut inflammation and HERVs during the inflammatory process, both before and after a diet with and without gluten. Data on inflammatory markers such as procalcitonin (PCT), C-reactive protein (CRP), the erythrocyte sedimentation rate (ESR), the monocyte distribution width (MDW), etcetera, will also be included. These data, together with other clinical information, could certainly be helpful to better understand the relationship between HERVs and gut inflammation. These studies should investigate the expression levels of HERVs in relation to the immune response to HERVs and the presence of anti-TG2 Abs both before and after a gluten-containing and gluten-free diet.

For these reasons, it is important to acknowledge the limitations of this study. Firstly, it was not possible to perform a simultaneous analysis of HERVs and INF-α gene expression, and the sample size was relatively small, which may affect the generalizability of the results. However, it is important to emphasize that the expression of HERVs is highly sensitive to many variables, including environmental, physiological, gut microbiota dysbiosis, and pathological factors such as stress. Therefore, despite these limitations, we believe our findings provide further support to the existing literature.

## 5. Conclusions

Our research has provided additional information on the potential role of HERVs and IFN-α in the immunopathology of CD. While these findings raise intriguing questions, there is a great deal more to investigate and comprehend in this continuously advancing field of research. The underlying molecular mechanism(s) responsible for these high levels of Abs against HERVs in patients with CD will be further investigated and elucidated with a larger cohort. In this way, we will be able to examine the complex interactions between genes and the environmental factors in patients with CD, which can be influenced and modified by HERVs through epigenetic mechanisms, such as DNA methylations, histone modifications, or miRNA expression modulation [7,79,80].

## Figures and Tables

**Table 1:** Demographic, clinical, and laboratory features of patients with celiac disease (CD) and the healthy controls (HCs).

	CD *n* = 40	HCs *n* = 41
Age, years	33 ± 21.59	31.4 ± 21.16
Female sex, n (%)	25 (62.5)	26 (65)
Anti-transglutaminase 2 (TG2) antibodies (U/mL), n (%)	35 (87.5)	-
Anti-endomysium Abs, n (%)	13 (32.5)	-
Anti-deamidated gliadin Abs IgA (U/mL), n (%)	19 (47.5)	-
Anti-deamidated gliadin Abs IgG (U/mL), n (%)	17 (42.5)	-

**Table 2:** The prevalence of age-related Abs in patients with CD and in the HCs. The number of individuals responsive to the single antigens are provided, with their relative percentages. The statistically significant values are highlighted in bold, while the values close to the threshold of statistical significance are shown with a star (*).

Age	N	Subject	INF-α	*p*	HERV-K	*p*	HERV-H	*p*	HERV-W	*p*
0–11	**10**	CD	8	**0.0300**	7	**0.0274**	6	0.0743 *	7	0.0062
			(80%)		(70%)		(60%)		(70%)	
	**12**	HCs	3		2		2		1	
			(25%)		(16.66%)		(16.66%)		(8.33%)	
12–18	**5**	CD	4	0.2063	3	0.5238	1	1.0000	2	1.0000
			(80%)		(60%)		(20%)		(40%)	
	**5**	HCs	1		1		1		1	
			(20%)		(20%)		(20%)		(20%)	
19–40	**8**	CD	6	**0.0406**	7	**0.0014**	3	0.5692	3	0.5692
			(75%)		(87.5%)		(37.5%)		(37.5%)	
	**7**	HCs	1		0		1		1	
			(14.28%)		(0%)		(14.28%)		(14.28%)	
41–51	**7**	CD	5	0.1319	4	0.1189	4	0.1189	4	0.1189
			(71.42%)		(57.14%)		(57.14%)		(57.14%)	
	**8**	HCs	2		1		0		0	
			(25%)		(12.5%)		(0%)		(0%)	
52–81	**10**	CD	6	0.0573 *	6	0.0573 *	3	0.2105	1	1.0000
			(60%)		(60%)		(30%)		(10%)	
	**9**	HCs	1		0		0		0	
			(11.11%)		(0%)		(0%)		(0%)	

## Data Availability

The original contributions presented in the study are included in the article, further inquiries can be directed to the corresponding author.
